# Attrition rates in hand and wrist trauma trials: a systematic review and meta-analysis protocol

**DOI:** 10.1186/s13643-026-03201-1

**Published:** 2026-06-12

**Authors:** Sophie Elisa Gibbs Ludbrook, Andrew James Robert Waters, Justin Conrad Rosen Wormald

**Affiliations:** 1 Adelaide University, Adelaide, SA Australia; 2 Department of Orthopaedics, Rheumatology and Musculoskeletal Sciences, Kadoorie Centre, Oxford Trauma and Emergency Care, Nuffield University of Oxford, Oxford, OX3 9DU UK

**Keywords:** Attrition, Hand trauma, Wrist trauma, Systematic review, Meta-analysis

## Abstract

**Background:**

Hand and wrist injuries are increasingly prevalent and impact individuals’ abilities to participate in society and generate income. Attrition in the hand and wrist trauma treatment time course and trials is a recognised issue, with patients disengaging from follow-up due to various logistical and socioeconomic barriers. While several studies have addressed this, the timeline and causes of attrition in trials remain inconsistent. This meta-analysis aims to evaluate the attrition rates among patients with hand and wrist trauma across all prospective randomised controlled trials (RCTs), identifying critical time points for attrition, at-risk patient populations, and comparing surgical versus non-surgical interventions.

**Methods:**

The databases Embase, PubMed, and CENRAL will be searched. Abstracts will be screened by two persons independently to identify eligible studies. The systematic review will include prospective randomised controlled trials in participants with hand and/or wrist trauma. The review will consider patient demographics and proportions of attrition rates at time frames of 30 days, 90 days, 6 months, and 12 months. The meta-analysis will compare attrition time frame considering patient demographics, intervention and injury types, and documented attrition reasons. The Cochrane risk-of-bias tool 2 will be used to assess the risk of methodological bias in the randomised controlled trials.

**Discussion:**

This systematic review and meta-analysis will address the critical gap in understanding attrition rates in hand/wrist trauma research. By identifying which patients are at risk and when attrition occurs, we aim to develop a toolkit to improve follow-up rates and, in turn, patient outcomes. Ultimately, the findings will inform future clinical practices and research methodologies, contributing to more representative and robust evidence in hand/wrist trauma management.

**Systematic review registration:**

PROSPERO CRD42024588638.

**Supplementary Information:**

The online version contains supplementary material available at https://doi.org/10.1186/s13643-026-03201-1.

## Background

Hands are the most common body site to present with injury and play a substantial role in individuals’ ability to participate in society and generate income [1–5]. Hand and wrist trauma can be complex and often requires treatment with time-sensitive, multidisciplinary multi-disciplinary follow up to ensure good outcomes and return to normal functioning [6].

Attrition in the treatment course is a recognised issue in musculoskeletal trauma [7]. Patients facing logistical and social challenges are more likely to disengage and not attend routine follow-up [8–11]. Low socioeconomic status, prioritisation of income over healthcare, logistical challenges of travelling great distances and administrative errors are commonly reported reasons for this [8, 9, 12, 13]. However, formal examination of associations between these reasons and attrition rate produces inconsistent results. The cause of this may be in part the populations studied, such as a general orthopaedic trauma population, or in part due to limitations to their methodology. In addition, there are a limited number of studies that have assessed the timeline of attrition rate in depth, such as loss at specific time points, and no meta-analyses have been performed [13–16].

The incidence of hand trauma is increasing, as is its societal impact. This increases the need for in-depth understanding of management, follow-up, and outcomes in the short and long term [17].

The proposed meta-analysis will analyse all prospective randomised controlled trials (RCTs) to assess the attrition rate of hand trauma participants in both surgical and non-surgical interventional studies. This will enable identification of when attrition occurs, clarify at-risk patient populations and compare differences in surgical and nonsurgical RCTs. This may provide a foundation for the development of an evidence-based tool kit to improve attrition rates in RCTs in this population. This aims to in turn reduce attrition and support connection throughout a patient’s hand trauma treatment course, ultimately benefiting patient outcomes.

## Methods/design

### Eligibility criteria and information sources

This protocol has been written using the PRISMA-P (preferred Reporting Items for Systematic review and Meta-Analysis Protocols) Checklist [18]. It has been registered on the PROSPERO International Prospective Register of Systematic Reviews [19].

The review will be a PRISMA-compliant systematic review and meta-analysis [20].

### Search strategy and selection criteria

The search strategy will be developed with the aim to assemble all articles relating to the above stated review objective. The search strategy will be applied to databases PubMed and Embase from inception until June 2024 alongside the Cochrane Central Register of Controlled Trials (CENTRAL). MeSH term and free-text search strategies will be refined using the terms stated below. Duplicate papers will be removed from the pool. Results of the search will be filtered using titles and abstracts by two authors (AW and SL). Papers with unrelated topics will be excluded. Papers left from this will be read in full and further screened by the two authors. The final papers’ reference lists will also be screened to catch any literature previously missed. Any inconsistencies in inclusion decisions will be moderated by third author (JW).

Citations will be managed in EndNote, and the data management and list of studies screened in Excel.

All prospective randomised controlled trials (RCTs) assessing hand and wrist trauma populations will be eligible for inclusion. Global papers published in any language will be included. We will use Google Translate to assess eligibility of non-English language studies.

Studies that have recorded attrition rates will have data extracted and categorised into time periods at 30 days, 90 days, 6 months, and 12 months. Through meta-analysis, the time frame during which attrition occurs alongside the method of medical intervention will be compared and evaluated. The study approach is designed utilising the guideline, Population, Intervention, Comparison, Outcome (PICO) Model for Clinical Questions [21].

#### Participants

Participants included will be patients of all ages and demographics with hand and wrist trauma who have been recruited into prospective RCTs of both surgical and non-surgical interventions.

#### Outcome

Outcome will be attrition–proportion of patients who are lost to follow-up at the aforementioned estimated time points (primary outcome). All studies reporting on relevant clinical outcomes will be included.

#### Study design

Prospective randomised controlled trials will be eligible.

Observational studies, letters, opinion pieces, and literature reviews will be excluded.

Papers with no data on attrition rate will be excluded.

There will be no limitations to study size or patient inclusion criteria.

### Data extraction

The approach to extraction and evaluation of data will be compliant with recommendations in the Cochrane Handbook of Systematic Reviews of Interventions [22]. The data will be processed by two authors (AW and SL) into a formatted electronic database/form. Decisions to include data cohorts in the review’s pool alongside the approach to categorisation of this will be overseen by another author (JW). Data that will be collected and compared will be each study’s elements and patient demographic, aetiology, diagnosis and management of the patients’ trauma, attrition rates and any recorded reason for this, time points of attrition and any outcome measures.

Where data provided in studies is categorised into anatomical location of trauma, this will remain distinguishable. Data only relevant to upper limb trauma, and location of injury not specified, study authors will be contacted by email, a maximum of two times if required. If no further details are provided the study will be excluded and reported accordingly in the PRISMA flow chart.

### Outcome measures

The primary outcome will be the attrition rates of individual studies, at the following time points: 30 days, 90 days, 6 months, and 12 months. We will calculate the proportion of patients lost to follow-up at these time points for each study.

### Risk of bias

Risk of bias for studies assessed with Cochrane risk-of-bias 2 (RoB 2) tool [23]. Through application of the PICO framework, we have established that suitable studies would fulfil the below:

**Table:** 

Participants:	No selection bias
Intervention and comparison:	Intervention and time course of follow up defined, no selection bias
Outcomes:	No loss of data, easily compared methodology in studies, clearly recorded follow up times, evidence results correspond to intended outcomes

Each study not fulfilling the points above will be evaluated individually and their bias risk established as moderate or higher. Any gaps in data provided will be attempted to be followed up.

### Data analysis and synthesis

We will perform simple descriptive statistics for patient demographics and the individual attrition rates for each study, reported as proportions. (%) of attrition in hand and wrist trauma patients will be calculated using pooled data collected from each study. Data will be appropriately categorised into time points. We will perform meta-analysis of proportions to determine pooled attrition rates across the included study populations. R version 4.0.3 (2020–10-10) will be used with the ‘meta’ (version 5.2–0) and ‘metafor’ (version 3.4–0) packages [24]. Statistical heterogeneity will be quantified for all direct comparisons using the I-squared statistic [25]. The meta-analysis results will be displayed in forest plots where possible with a funnel plot to assess publication bias in our primary outcome [26].

We will perform subgroup analyses or studies of surgical versus non-surgical interventions, to determine whether there are different attrition rates depending on the type of intervention. We will also perform subgroup analyses for hand versus wrist injuries to determine differences in attrition.

### Confidence in cumulative evidence

Recommendation based on evidence used by the GRADE Approach.

## Discussion

The burden of hand and wrist trauma is increasing globally [27]. There has been no meta-analysis of attrition rate in hand and wrist trauma conducted before. Current published literature has limited population sizes and published mixed conclusions of patients at risk of attrition rate. There is also limited published literature addressing the exact time course of attrition. We hope to identify suitable RCTs and extract applicable data to help evaluate and understand attrition rates following hand trauma. By conducting a meta-analysis, we will clarify inconsistencies in current literature and be able to better understand at-risk patient subpopulations, the timeline and burden of attrition rate. In addition, we will directly compare attrition rates of patients receiving surgical and nonsurgical interventions at different time points.

The acceptable attrition rate in RCTs, to minimise concern of bias, is less than 20% [28]. Actual attrition rates in RCTs assessing hand and wrist trauma are recorded between 19 and 42% [11, 13, 29]. Patients lost to follow-up are often excluded in RCTs.

If attrition rates are consistently high in certain sub-populations, then the conclusions drawn from clinical trials are at risk of missing up to almost half of their originally intended patient sample and in turn, misinterpreting patient outcomes and bias.

This may consequently produce a low fragility index, risking misrepresentation of actual outcomes in the broader population. The fragility index assists to assess the robustness of results and conclusions drawn in clinical trials [30]. A key component of the fragility index is the attrition rate, which if high, can lower a trial’s fragility index [31].

Through conducting a meta-analysis, we seek to understand which patients are lost to follow-up, and where possible, when and why. Upon understanding this subpopulation, through an RCT we aim to develop and implement a toolkit catering for these patients who are most at risk, aiming to reduce attrition rate to an acceptable level. If attrition rate is reduced, and a larger range of patient subgroups can be followed up, we can seek to understand treatment outcomes with less bias. In turn, conclusions drawn will better represent the broader population and be of use in clinical decision making.

The conclusions we draw will have solid foundational methodology and approach. Studies included in the review and analysis will be RCTs. It is also expected that the volume of data for hand trauma will be limited. Hence, we have expanded the search to include wrist trauma. We understand that this may limit the review, so we will ensure delineation from hand and wrist trauma patients to assist in the analysis of each sub-population of the data pool. Any bias encountered and possible influence on conclusions drawn will be assessed and discussed.

We aim to better understand attrition rates in hand trauma RCTs—who these patients are, when they are at most risk of attrition and, in addition, compare surgical and non-surgical treatment courses. This will inform how we approach treatment of hand trauma patients at risk of attrition. Current literature supports the concept of identifying patients at risk of attrition and encourages working to maintain patient contact [11, 13]. There is however no formal guidelines or tool to apply to patient follow-up. Once performed, we hope the systematic review and meta-analysis results will assist to aid discussion and recommendations to guide future research in this topic. Ultimately, we hope to conduct experimental clinical trials to develop a tool to assist in identifying and addressing the anticipated burden of attrition in hand trauma patients.

## Supplementary Information


Supplementary Material 1.

## Data Availability

The datasets used and/or analysed during the current study are available from the corresponding author upon reasonable request.

## Abbreviations

NICE: National Institute for Health and Care Excellence
PRISMA: Preferred Reporting Items for Systematic review and Meta-Analysis Protocols
PROSPERO: International Prospective Register of Systematic Reviews
RCT: Randomised controlled trials
RoB: Risk of bias
