# Supplementary material for: Attrition rates in hand and wrist trauma trials: a systematic review and meta-analysis protocol
Source: Syst Rev. 2026 Jun 12;15:236. doi: 10.1186/s13643-026-03201-1 (PMC13523474; doi:10.1186/s13643-026-03201-1)
Supplement: Supplementary file 1 [file 13643_2026_3201_MOESM1_ESM.docx]

**Search Strategy**

The search strategy was developed with the aim to assemble all articles relating to the stated review objective. The search strategy will be applied to databases Pubmed and Embase from inception until June 2024 alongside the Cochrane Central Register of Controlled Trials (CENTRAL). MeSH term and free-text search strategies will be refined using the terms stated below.

Embase

1. Injury segment

Exp *hand injury OR exp *wrist injury OR ((hand* OR wrist* OR distal ulna OR distal radius) adj3 (injur* OR fractur* OR trauma)).ti,ab

2. Attrition segment

follow up.sh OR Patient dropout.sh OR (attrition OR follow up OR followup OR missed visit* OR "failure to return" OR "failed to return" OR "fail to return" OR "fail to complete" OR "failed to complete" OR "failure to complete" OR drop-out* OR dropout* OR dropped out).ti,ab

3. RCT segment

((exp randomized controlled trial/ OR controlled clinical trial/ OR random$.ti,ab. OR randomization/ OR intermethod comparison/ OR                   placebo.ti,ab. OR (compare OR compared OR comparison).ti OR ((evaluated OR evaluate OR evaluating OR assessed OR assess) AND (compare OR compared OR comparing OR comparison)).ab. OR (open adj label).ti,ab. OR ((double OR single OR doubly OR singly) adj (blind OR blinded OR blindly)).ti,ab. OR double blind procedure/ OR parallel group$1.ti,ab. OR (crossover OR cross over).ti,ab. OR ((assign$ OR match OR matched OR allocation) adj5 (alternate OR group$1 OR intervention$1 OR patient$1 OR subject$1 OR participant$1)).ti,ab. OR (assigned OR allocated).ti,ab. OR (controlled adj7 (study OR design OR trial)).ti,ab. OR (volunteer OR volunteers).ti,ab. OR human experiment/ OR trial.ti.)) NOT ((((random$ adj sampl$) adj7 ("cross section$" OR questionnaire$1 OR survey$ OR database$1)).ti,ab. NOT (comparative study/ OR controlled study/ OR randomi?ed controlled.ti,ab. OR randomly assigned.ti,ab.)) OR (cross-sectional study/ NOT (exp randomized controlled trial/ OR controlled clinical trial/ OR controlled study/ OR randomi?ed controlled.ti,ab. OR control group$1.ti,ab.)) OR (((case adj control$) AND random$) NOT randomi?ed controlled).ti,ab. OR (systematic review.ti,ab. NOT (trial.ti. OR study.ti)) OR (nonrandom$ NOT random$).ti,ab. OR "random field$".ti,ab. OR (random cluster adj3 sampl$).ti,ab. OR ((review.ab. AND review.pt.) NOT trial.ti.) OR ("we searched".ab. AND (review.ti. OR review.pt.)) OR "update review".ab. OR (databases adj4 searched).ab. OR ((rat OR rats OR mouse OR mice OR swine OR porcine OR murine OR sheep OR lambs OR pigs OR piglets OR rabbit OR rabbits OR cat OR cats OR dog OR dogs OR cattle OR bovine OR monkey OR monkeys OR trout OR marmoset$1).ti. AND animal experiment/) OR (animal experiment/ NOT (human experiment/ OR human/)))

Pubmed

1. Injury segment

"Hand Injuries"[mh] OR "wrist injuries"[mh] OR hand trauma[tiab] OR hand injur*[tiab] OR wrist trauma[tiab] OR wrist injur*[tiab] OR hand fracture*[tiab] OR wrist fracture*[tiab] OR distal ulna fracture*[tiab] OR distal ulna injur*[tiab] OR distal ulna trauma[tiab] OR distal radius fracture*[tiab] OR distal radius injur*[tiab] OR distal radius trauma[tiab]

2. RCTs segment

(randomized controlled trial [pt] OR controlled clinical trial [pt] OR randomized [tiab] OR placebo [tiab] OR clinical trials as topic [mesh:noexp] OR randomly [tiab] OR trial [ti]) NOT (animals [mh] NOT humans [mh])

3. Attrition segment

"patient dropouts"[mh] OR Attrition[tiab] OR "follow up"[tiab] OR missed visit*[tiab] OR "failure to return"[tiab] OR failed to return[tiab] OR fail to return[tiab] OR fail to complete[tiab] OR failed to complete[tiab] OR failure to complete[tiab] OR drop-out*[tiab] OR dropout*[tiab] OR dropped out[tiab]

Cochrane Library

Injury

[mh "Hand Injuries"] OR [mh "wrist injuries"] OR ("hand trauma" OR (hand NEXT injur*) OR "wrist trauma" OR (wrist NEXT injur*) OR "distal radius" OR "distal ulna" OR (hand NEXT fracture*) OR (wrist NEXT fracture*)):ti,ab

RCTs

(limit to trials)

Duplicate papers will be removed from the pool. Results of the search will be filtered using titles and abstracts by two authors (AW and SL). Papers with unrelated topics will be excluded. Papers left from this will be read in full and further screened by the two authors. The final papers’ reference lists will also be screened to catch any literature previously missed. Any inconsistencies in inclusion decisions will be moderated by third author (JW).

Citations will be managed in EndNote, and the data management and list of studies screened in Excel.
